# Light field–controlled PHz currents in intrinsic metals

**DOI:** 10.1126/sciadv.adv5406

**Published:** 2025-06-25

**Authors:** Beatrix Fehér, Václav Hanus, Weiwei Li, Zsuzsanna Pápa, Judit Budai, Pallabi Paul, Adriana Szeghalmi, Zilong Wang, Matthias F. Kling, Péter Dombi

**Affiliations:** ^1^ HUN-REN Wigner Research Centre for Physics, Konkoly Thege M. út 29-33, 1121 Budapest, Hungary.; ^2^ Physics Department, Ludwig Maximilians Universität, Geschwister-Scholl-Platz 1, 80539 München, Germany.; ^3^ Max Planck Institute of Quantum Optics, Hans-Kopfermann-Straße 1, 85748 Garching, Germany.; ^4^ ELI-ALPS Research Institute, Wolfgang Sandner utca 3, 6728 Szeged, Hungary.; ^5^ Institute of Applied Physics, Friedrich Schiller University Jena, Albert Einstein Str. 15, 07745 Jena, Germany.; ^6^ Fraunhofer Institute for Applied Optics and Precision Engineering, Centre of Excellence in Photonics, Albert Einstein Str. 7, 07745 Jena, Germany.; ^7^ SLAC National Accelerator Laboratory, 2575 Sand Hill Rd, Menlo Park, CA 94025, USA.; ^8^ Department of Applied Physics, Stanford University, 348 Via Pueblo, Stanford, CA 94305, USA.

## Abstract

Oriented electric currents in metals are routinely driven by applying an external electric potential. Although the response of electrons to the external electric fields occurs within attoseconds, conventional electronics do not use this speed potential. Ultrashort laser pulses with controlled shapes of electric fields that switch direction at petahertz frequencies open perspectives for driving currents in metals. Light field–driven currents were demonstrated in various media including dielectrics, semiconductors, and topological insulators. Now, our research question is whether we can drive and control orders of magnitude more charge carriers in metals enabling ultrafast switching with practically low-energy, picojoule-level pulses. Here, we demonstrate the interaction of light with nanometer-thick metallic layers, which leads to a generation of light field–controlled electric currents. We show that the implantation of metallic layers into a dielectric matrix leads to up to 40 times increase of the sensitivity in contrast to a bare dielectric, decreasing the intensity threshold for lightwave electronics.

## Teaser

PHz-frequency currents are generated and controlled in metals by femtosecond laser light.

## INTRODUCTION

When laser pulses interact with solid materials, electrons are driven across energy bands and system boundaries and along various dispersion curves. Quantum control of electron trajectories, e.g., with two-color fields (*1*, *2*), or asymmetric strong fields just below the material’s damage threshold enable rectification of the optical field to directed electronic currents. In the latter case, a wide bandgap material can undergo a transient metallization (*3*) and electronic response to the optical field becomes immediate, enabling petahertz (PHz) switching frequency of the current direction (*4*, *5*). The ultrafast nature of these currents of PHz frequencies has been evidenced by the observation of high-harmonic generation (HHG) (*6*, *7*) or by rectification of asymmetric few-cycle strong fields (*4*). The light field asymmetry is usually controlled by stabilizing the so-called carrier-envelope phase (CEP) of few-cycle laser pulses. Using CEP-stabilized laser sources, the light field control over the PHz currents has been achieved in various media such as gases (*8*), wide bandgap materials as dielectrics (*4*, *9*, *10*), semiconductors (*10*–*12*), and two-dimensional (2D) materials (*13*–*15*). The progress in this field of lightwave electronics inspires ideas for PHz optoelectronic components such as logic gates, memories, or waveguide integrations (*12*, *16*, *17*).

To develop useful PHz devices, the sensitivity to the driving field needs to be enhanced. A viable approach is to drive currents directly in metals, where the electrons are readily available in the conduction band, potentially increasing the number of electrons exposed to the driving light field. The nonlinear behavior, which is essential for the asymmetric response, is then defined by the trajectories the electrons follow in the conduction band within the Brillouin zone (BZ) (*18*, *19*).

Recent observation of PHz currents in n-type semiconductors directly (*13*) or by means of the detection of high harmonics (*20*) hints at the fact that the light field control of ultrafast currents in classical metals with rich electron population in conduction bands is within reach. That said, the demonstration of light field control in metals at picojoule pulse energy level could open avenues for the design of PHz switching devices.

Here, we realize laser-field current generation and control in metals using a 2D-layered (nanolaminate) material. We use the CEP of a few-cycle laser pulse to drive currents within a volume enclosed by gold electrodes connected to an external circuit. Our samples comprise alternating layers of Ir metal and Al_2_O_3_ dielectric, fabricated using atomic layer deposition (ALD) (*21*). We validate our findings with samples containing single nanolayers of Ir and Au coated with Al_2_O_3_. Previous works using low-pulse-energy lasers to drive CEP-dependent currents in nonmetallic systems (*10*, *12*, *14*) relied on lock-in detection due to unspecified background noise. Here, we show that the background noise in these metallic samples is low enough for the modulated CEP signal to stand out, making it directly observable in an oscilloscope trace. The Ir nanolaminate has previously showed its applicability in a CEP-scanning device for characterizing few-cycle laser beams (*22*). Furthermore, it exhibits a high third-order nonlinear susceptibility (*23*, *24*). In relation to the measured current yield, we establish a straightforward empirical relationships linking the measured current, metal layer thickness, and the third-order nonlinear susceptibility. Consequently, we show that the current yield is proportional to the total number of electrons in the system. Finally, we discuss the relevant mechanisms of the current excitations in metals.

## RESULTS

### Experimental setup

**Fig. 1.**
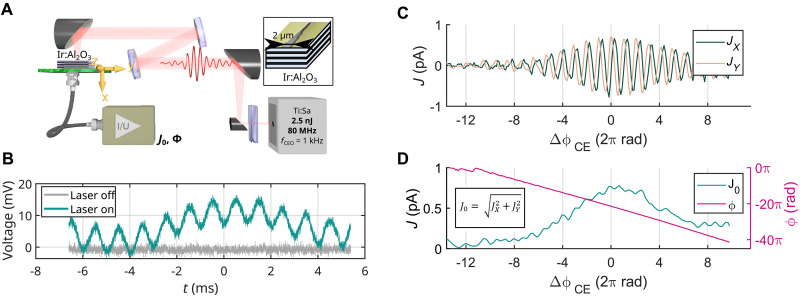
Experimental setup. (**A**) A phase-stabilized laser beam from a Ti:sapphire laser oscillator is led through dispersion compensation elements (fused silica glass wedges) to ensure the shortest pulse duration on target. An off-axis parabola telescope was used to expand the beam and an off-axis parabola mirror to focus it on the Ir:Al_2_O_3_ nanolaminate. The oscillator is operated at the carrier-envelope offset frequency, $f_{\text{CEO}}=$ 1 kHz. As the CEP of pulses and subsequently the induced ultrafast current oscillate at this frequency, we can detect the CEP-dependent current magnitude $J_{0}$ and phase ϕ with a lock-in amplifier. Inset shows the nanolaminate structure of the sample: alternating metallic and Al_2_O_3_ layers, together with the shape of the electrodes. In case of sample with ALD cycle number # = 64, the thickness of Ir and Al_2_O_3_ is 3.8 and 3.2 nm, respectively, and there is 25 repetitions in the stack, reaching 175 nm total thickness of the nanolaminate stack. (**B**) Output of the transimpedance preamplifier measured with an oscilloscope: Laser is on and it illuminates the center of the junction, or laser is off. The $f_{\text{CEO}}$ modulation is visible. (**C**) $J_{x}$ and $J_{y}$ lock-in current as a function of $\Delta \phi_{\mathrm{CE}}$ , fine-tuned by the adjustment of fused silica wedges. (**D**) Photocurrent $J_{0}$ , and ϕ , the lock-in phase, as a function of $\Delta \phi_{\text{CE}}$ , fine-tuned by the wedges.

We examine the generation of CEP-dependent ultrafast currents in metallic layers in a setup based on a phase-stabilized Ti:sapphire laser oscillator (see Fig. 1A) (*10*). The key principle is to expose the generating medium to the few-cycle laser pulses with well-defined variation of CEP. As for some CEP the electric field waveforms are asymmetric, the electronic reaction in the medium can lead to an oriented current (*4*, *10*, *12*). The laser pulse is defined as

$$E\left(t\right)=E_{0}eg\left(t\right)\text{cos}\left(\omega t+\phi_{\text{CE}}\right)$$ (1)

where *g*(*t*) is the envelope, $E_{0}$ is the amplitude of the electric field, e is the unitary vector of the polarization direction, and $\phi_{\text{CE}}$ is the CEP.

The light field–driven current *J* dependent on $\phi_{\text{CE}}$ can be detected by placing metallic electrodes on the surface next to the interaction volume, which are connected to a current preamplifier that converts the current to voltage. In Fig. 1B, we show the voltage from the preamplifier (gain, 10^9^ V/A; FEMTO Messtechnik GmbH, DLPCA-200) acquired with an oscilloscope without postprocessing. The modulation of the CEP at $f_{\text{CEO}}$ = 1 kHz is clearly visible in the voltage trace, showing about 20-dB signal-to-noise ratio. The $f_{\text{CEO}}$ frequency is the frequency at which the CEP of the pulses in the laser pulse train oscillates. $f_{\text{CEO}}$ is set in the laser oscillator. Such a trace measurement requires a proper grounding to reduce noise, which is a rather lengthy procedure. That is why we rather stick to a lock-in detection throughout this paper as we switch samples frequently. In the case of lock-in detection, the in-phase $J_{x}$ and quadrature $J_{y}$ currents are then a measure of the magnitude of the CEP-induced current oscillation that is in phase and out of phase with the $f_{\text{CEO}}$ reference clock, respectively.

We confirm the CEP sensitivity of metallic nanolaminates with a wedge scan. If such a pulse passes through a pair of wedges, it undergoes a CEP shift depending on the amount of glass. Since the phase velocity and group velocity is different in glass, we can shift the CEP of the pulse by adding glass material to the beam path. As a result, the measured $J_{x}$ and $J_{y}$ oscillate as a function of the amount of glass added as the phase of the oscillation of $\phi_{\text{CE}}$ detunes from the lock-in reference (see Fig. 1C). As the laser pulse is stretched, the CEP effect weakens and the amplitude of the oscillations decreases. We then define $J_{0}^{2}≔J_{x}^{2}+J_{y}^{2}$ , together with $\phi ≔\text{arctan}\left(J_{y}/J_{x}\right)$ (see Fig. 1D). The former allows us to assess the magnitude of the CEP effect, and the latter is a direct measure of the CEP change from one wedge position $\phi_{\text{CE},1}$ to another $\phi_{\text{CE},2}$ , i.e., $\phi \equiv \Delta \phi_{\text{CE}}=\phi_{\text{CE},2}-\phi_{\text{CE},1}$ . Hence, $J_{0}$ and ϕ of the current from the sample are measures of the magnitude and phase of the CEP-dependent ultrafast current in the medium.

The amplitude of the electric field at the sample, $E_{0}$ , is set to $0.3<E_{0}<0.6$ V/Å. The lower limit corresponds to 500 pJ pulse energy. The range of examined $E_{0}$ values was limited by the noise floor in the $J_{0}$ measurement (about 50 fA) and the laser pulse energy. We choose to refer our observations to the intensity at the surface of samples for two reasons: (i) the system is sensitive only to currents driven in top layers; (ii) a single metal layer is subwavelength and a few nanometers thick, which is shorter than the characteristic penetration depth of the field in the Ir metal. See the Supplementary Text for details.

The interaction conditions can be further characterized with Bloch frequency $\omega_{B}={\mathrm{eE}}_{0}a_{\text{Ir}}/\hbar$ and dynamic localization parameter $\gamma_{\text{DL}}=\omega_{B}/\omega_{0}$ . Here, $a_{\text{Ir}}=0.387$ nm is the lattice constant of Ir. Under our experimental conditions, we reach intensities for which $\omega_{B}=4.11\times 10^{15}$ rad/s; hence, it is higher than the laser frequency $\omega_{0}=2.35\times 10^{15}$ rad/s, yielding dynamic localization parameter $\gamma_{\text{DL}}=1.8$ . When this value is higher than 1, the electron wave packets are localized by coupling between the neighboring lattice sites and the electrons can be treated as classical particles that follow Bloch’s acceleration theorem. Within this approximation, the highly nonlinear motion of the electrons, such as Bloch oscillations, has been theoretically identified (*25*, *26*). It has been shown earlier (*27*) that the presence of Bloch oscillations can lead to clipping of current oscillations, and the consequent nonlinear response can lead to a nonzero transferred charged, i.e., the appearance of a DC current component once the driving laser field is over.

The samples under investigation are 2D nanolaminates prepared with an atomic-layer deposition method. During the deposition, metallic Ir deposition cycles were alternated with Al_2_O_3_ cycles. By tuning the number of cycles, various combinations of layer thicknesses are achieved, and consequently, different metallic content volume fractions *f* are manufactured. The number of cycles for Al_2_O_3_ is fixed to 35, while the number of cycles of Ir is #={0,8,16,32,64} , which leads to f={0%,13%,23%,38%,55%} fractional content, respectively. Thus, individual nanolayers of Ir metal have thickness $d_{1}=\left\{\;0,0.24,0.48,0.96,1.92,3.84\;\right\}$ nm. The deposition of Ir and Al_2_O_3_ single nanolayers is repeated numerous times to form several hundred nanometer-thick slabs. The detailed investigation of the sample properties was carried out elsewhere (*21*). The relevant findings for this work are that the Ir percolates rather fast and forms homogeneous layers rather than nanoparticles. The presence of a Bragg peak in the x-ray reflectivity measurements evidenced the continuous metallic layers in the samples (*21*) [batches of samples presented in this work are identical to those investigated by Paul *et al.* (*21*)]. Ir nanolayers are considered to have a grain size of about 30 nm (*28*).

After the deposition of a pair of gold electrodes, a micrometer-sized gap is formed. As the cap layer is formed by Al_2_O_3_, the samples appear as nonconductive, when resistance is measured across the electrodes (*R* > 10^6^ ohms). Additionally, we also examined a sample with a 3.5-nm-thick layer of Au with 20-nm Al_2_O_3_ capping, and a single-layer sample with 1.8-nm-thick Ir and 3.15-nm-thick Al_2_O_3_ capping layer.

To accurately characterize all samples with different metallic content in terms of current generation, it is crucial to first fix the interaction conditions and then to minimize the effects of the illumination geometry. To ensure equal experimental conditions for all Ir samples, the measurements were executed during 1 day with the following parameters: laser beam power $P_{\text{avg}}$ = 0.12 W, focal spot size at full width at half maximum (FWHM) $w_{\text{FWHM}}$ = 1.8 μm, and pulse duration $\tau_{\text{FWHM}}$ = 5.5 fs at 80-MHz repetition rate. These settings lead to the maximum available electric field amplitude just above the sample: $E_{0}$ = 0.6 V/Å.

The light is polarized in a way that the polarization vector points from one electrode to the opposite one. The measurement of CEP-dependent ultrafast currents is known to exhibit a dependence on the size of the gap of the sensing electrodes (*10*). Therefore, we designed electrode shapes in a way that several gap sizes are available close to each other and they form five segments, each 5 μm long, so that the focused beam would fit well into each segment (see sample detail in Fig. 1A). To obtain a representative value for the current $J_{0}$ , we performed a 3D scan around the laser focus probing all the gaps and several lateral and axial positions (see Materials and Methods and Fig. 7 for an exemplary 3D scan). Finally, the maximum attained current $J_{0,\text{max}}\left(f\right)$ was noted for each sample. Thus, $J_{0,\text{max}}$ is a geometry-independent quantity.

### Ultrafast currents in metals

**Fig. 2.**
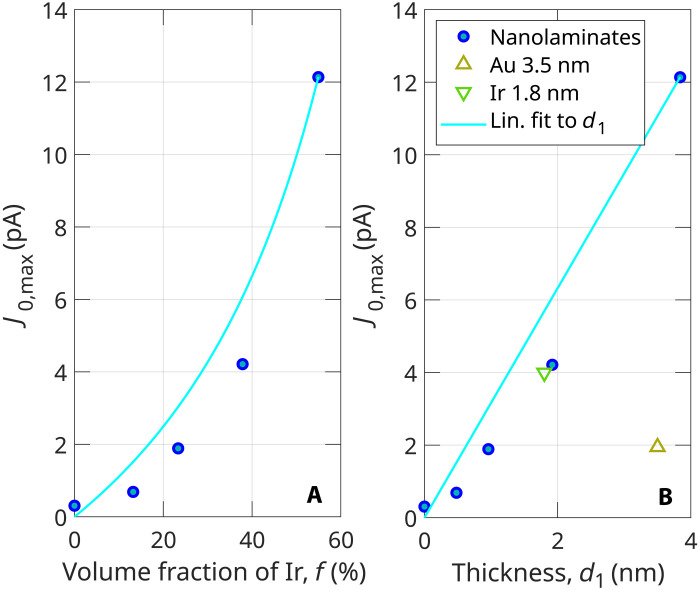
Ultrafast currents in metals as a function of volume fraction and Ir thickness. (**A** and **B**) Blue dots: Measured maximum current as a function of the volume fraction of Ir metal *f* and single-layer thickness $d_{1}$ , respectively. Each blue dot indicates a sample with different volume fraction. (B) Yellow triangle: $J_{0,\text{max}}$ of 3.5-nm Au capped with 20-nm Al_2_O_3_ layer. Green triangle: Single-layer sample with 1.8-nm Ir thickness. Cyan line: Linear fit to single-layer thickness $d_{1}$ : $J_{0,\text{max}}=3.2\;d_{1}$.

Our investigation of ultrafast currents as a function of the metallic volume fraction *f* in the 2D nanolaminates shows a clear trend: The higher is the fraction, the higher is $J_{0,\text{max}}$ (see Fig. 2A). Consequently, the presence of the metallic layers increased the ultrafast current gain compared to the Al_2_O_3_ reference sample with no metallic content 40 times.

This is achieved at a moderate field strength of 0.5 V/Å that is available with high–repetition rate laser oscillators that attain increased attention in the investigation of highly nonlinear optical phenomena (*22*, *29*–*34*). Furthermore, the dependence of current on the single-layer thickness, $d_{1}$ , is close to linear. We show it by a linear fit to the data with a cyan line in Fig. 2B. This indicates that the increase of the current might be related to the total number of conduction band electrons available in the system.

The current $J_{0}$ in the electric circuit can be understood as a consequence of the Ramo-Shockley theorem that has been already successfully applied to explain a nonlocal transfer of currents from light field–driven medium to an electric circuit (*8*, *35*). It states that the current $J_{0,\text{max}}$ is determined by Qv , where *Q* is the net charge moving at velocity *v*. Thus, in the case of metals, the value of the velocity appearing in this formula would be close to the Fermi velocity $v_{F}$.

Moreover, in charge-neutral periodic systems, the displaced charge *Q* has to be substituted by the net polarization vector P , representing a charge per unit surface. It is the action of the asymmetric laser field that brings the material to a state of nonzero polarization. Finally, with respect to the $J_{0,\text{max}}\left(d_{1}\right)$ dependence, we conclude that thicker the nanolayer, the more charge *Q* is displaced in the system, according to $Q=|\;P\;|d_{1}w_{\text{FWHM}}$ . Whence, this is in alignment with the close-to-linear dependence of $J_{0,\text{max}}\left(d_{1}\right)$ (see Fig. 2B). The discussed charge displacement does not exceed the grain boundaries of the polycrystal. It is in the order of units of lattice sites. Assuming unidirectional motion during the 5.5-fs duration of the laser pulse, moving with $v_{F}$ , the electrons reach a maximum of 4-nm distance corresponding to about 10 lattice sites. Similar short-range transport effects have been already investigated for the HHG process (*36*) where the localization and transport effects were identified to be responsible for features in the HHG emission spectrum.

This yield enhancement is not only a domain of the multilayer (nanolaminate) Ir system. In the single-nanolayer Au sample, we also observe a 13-fold enhancement of current compared to a bare sample under the same illumination conditions (see Fig. 2B). The measurements on a single Ir layer sample composed of a 30-cycle Ir (1.8 nm) layer, which is capped with a 35-cycle Al_2_O_3_ (3.15 nm) layer, yield a similar value of $J_{0,\text{max}}$ as the multilayer sample with 32 cycles of Ir. This finding suggests that the top metal layers play the major role in the formation of the measured CEP-dependent current. Consequently, the nonconductive layer of Al_2_O_3_ on the top acts only as an ohmic insulation between the Ir layer and Au electrodes.

The increase of the metal layer thickness beyond the tested samples might increase the current yield further until the point of saturation as the E-field is attenuated due to the skin effect in metals. However, in this experiment, we did not reach this limit (calculated as 19.2 nm for Ir; see Supplementary Text) as the thickest sample contains a 3.8-nm Ir layer. Experimenting with a thicker layer was hindered by technical issues, for example, fragility of the nanolaminate layer during the process of wire bonding.

**Fig. 3.**
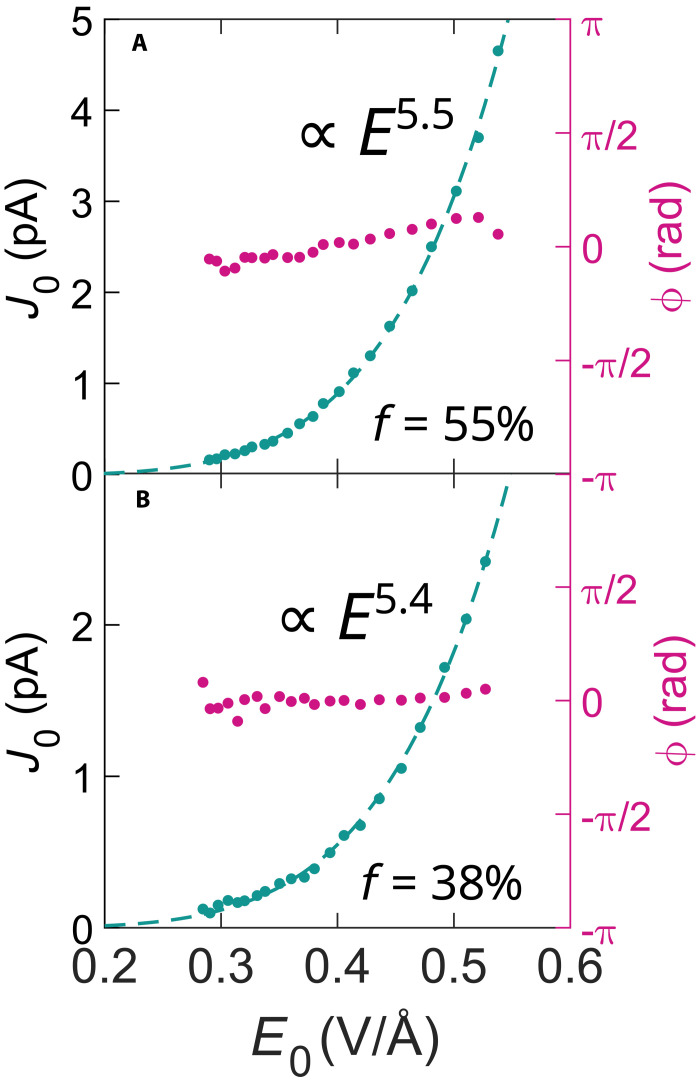
Ultrafast currents as a function of electric field strength. Green: Measured current $J_{0}$ as a function of the electric field amplitude $E_{0}$ . Magenta: Measured phase ϕ . (**A**) Sample containing 64 cycles of Ir ( f=55% ). (**B**) Sample of 32 cycles of Ir ( f=38%).

The detectable current $J_{0}$ emerges above the noise levels at focal field strengths about 0.3 V/Å, which correspond to a pulse energy of 500 pJ. The scaling of current $J_{0}$ as a function of the electric field amplitude $E_{0}$ follows a power law with an exponent of about 5.5 (see Fig. 3) (same exponent is obtained also for the Au sample, see Materials and Methods for the details). At the same time, the phase ϕ of the current is largely electric field independent over the investigated intensity range (see Fig. 3). This highlights the exceptional properties of metallic nanolayers for light field control applications and marks them being suitable for CEP detecting applications as, for example, spatial 3D CEP scanning (*22*) or for nonlinear photoconductive sampling (*13*).

## DISCUSSION

### Discussion about the origin of currents

Here, we would like to elaborate on possible mechanisms of the ultrafast current generation in metals. For this, let us recall the concepts of how the currents are generated in dielectrics and semiconductors (*4*, *12*, *14*). We will discuss the case when the energy of photon, ℏω , is lower than the bandgap $E_{g}$ . In the perturbative regime, characterized by Keldysh γ>1 (*37*, *38*), coherent superposition of out-of-phase multiphoton electronic excitation can result in an asymmetric population in *k*-space, thus directed current. Such current control schemes were shown earlier (*1*, *2*) and are understood as slower than the field oscillations, as the transition time is higher than the light field period (*39*). However, for higher intensities and wider band gaps, γ<1 , the multi-photon transition times become faster and the electronic dynamics can respond promptly to the field oscillations. In this case, the coupling of filled valence and empty conduction band states across one or multiple lattice sites induces electron population on virtual energy levels, which exists only when the field is present (*40*). The population of the virtual states oscillates together with the laser field oscillations. The mediators of these oscillations over virtual energy levels are called virtual carriers (VCs). If the CEP is such that the electric field of the laser pulse is asymmetric and the polarization response is nonlinear, the material can be left behind with a residual polarization that is then detected in the experiment.

**Fig. 4.**
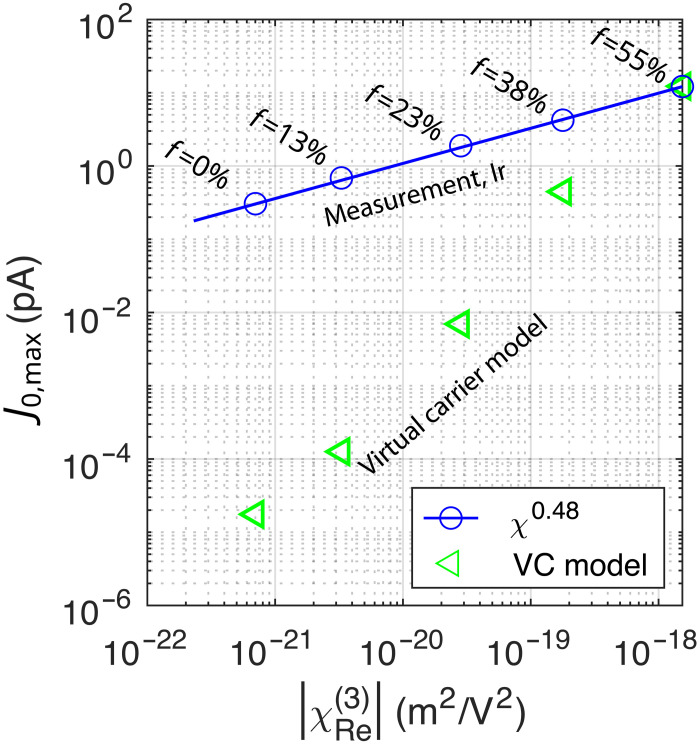
Discussion about the origin of currents. Blue: The maximum measured current $J_{0,\text{max}}$ as a function of the measured $\chi^{\left(3\right)}$ third-order nonlinear susceptibility. The labels at circles indicate the different samples with different Ir content. Blue line: A power-law fit is shown with a blue line to find the relationship between $J_{0,\text{max}}$ and $\chi_{\text{Re}}^{\left(3\right)}$ . Green triangles: Evaluation of a model based on virtual carriers (VC model) due to the interaction between the valence and conduction band in dielectrics. The model takes $\chi_{\text{Re}}^{\left(3\right)}$ as a parameter.

A model based on VCs for dielectrics and semiconductors (VC model) was developed (*41*) and successfully applied for semiconductors and dielectrics (*10*, *12*). The model provides a scaling, where the detected current can be expressed as a function of the nonlinear third-order susceptibility $\chi^{\left(3\right)}$ (*41*). The direct link to $\chi^{\left(3\right)}$ is advantageous since, as a macroscopic quantity, the nonlinear susceptibility can be independently measured. We expect that the metallic 2D nanolaminates have an exceptionally high $\chi^{\left(3\right)}$ (*23*, *24*). To confront the model, we performed a Z-scan measurement to obtain $\chi^{\left(3\right)}$ of our samples [the $\chi^{\left(3\right)}$ measurement was performed with 780-nm wavelength laser system delivering pulses of 70-fs duration; the Z-scan allows to extract the susceptibility indirectly from power transmitted through an aperture while the sample is translated along the optical axis through the laser beam focus; see the Supplementary Text for details on the measurement method]. The measurement of our samples yields an increasing nonlinear susceptibility as a function of the volume fraction, $\chi_{\text{Re}}^{\left(3\right)}\left(f\right)$ , and we identify an empirical relationship: $J_{0,\text{max}}\propto \sqrt{|\chi_{\text{Re}}^{\left(3\right)}|}$ (see Fig. 4). The values of $J_{0,\text{max}}$ are evaluated from VC model based on the measured $\chi_{\text{Re}}^{\left(3\right)}$ (see Fig. 4) [for the formula, see (*10*), equation 1]. We can see a strong discrepancy as the VC model predicts much steeper dependence on $\chi_{\text{Re}}^{\left(3\right)}$ . Moreover, the VC model does not reproduce the $J_{0,\text{max}}\left(E_{0}\right)$ scaling well either (see the Supplementary Text for the scaling evaluation). Since the model in question is based on the interaction between the valence and conduction bands, we suspect that the intraband motion might play an important role in the case of metals. We elaborate more on this scenario below.

**Fig. 5.**
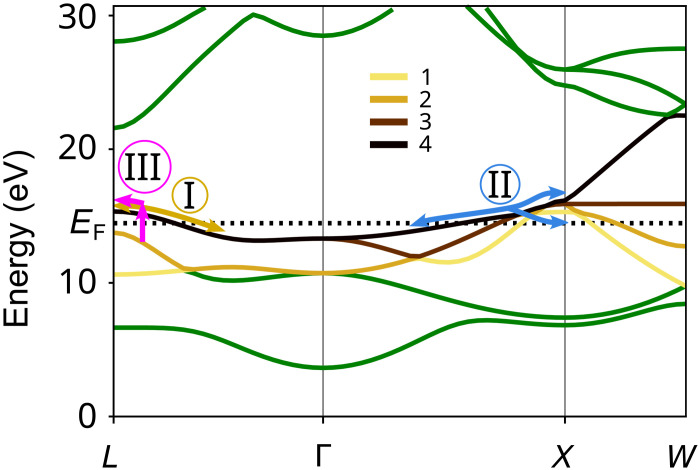
Band structure and Fermi level (dotted line) of a bulk Ir metal. It is calculated with a GPAW code using density functional theory (DFT). Labeled with numbers 1 to 4 are the bands that contain the Fermi surface. $E_{F}$ is depicted with the dotted line. Circles with I, II, and III show the light field–driven electron pathways discussed in the “Discussion about the origin of currents” section.

In Fig. 5, we show the calculated band structure obtained with density functional theory (DFT) calculation, a GPAW code (*42*–*44*). First, it is noteworthy to realize that valence-to-conduction band transitions are not available in Ir metal. Yet, band crossings and low energy vertical transitions of high momentum electrons are available between the conduction bands (see the arrows in Fig. 5). Second, the Fermi level is relatively high; specifically, the edges of the Fermi surface are beyond the half of the BZ length.

In simpler systems such as graphene, where the interaction of multiple conduction bands is turned off, the pathway of the field-driven population of electrons can be tracked from the measurement of power scaling. In the previous study of ultrafast currents (*45*), the power scaling exhibited more features as the measured residual current depended nonmonotonically on the laser field strength. However, in our case and observations elsewhere—which feature materials with more complex band structure (*4*, *9*, *10*, *12*)—we observe only monotonous increase of the current with only little change of the phase dependence. This could be due to the fact that the multiple involved pathways average out or the experiments do not reach the required intensity range to render some features observable (*39*).

Among the various pathways that field-driven electrons can follow (see the visualization in Fig. 5), let us highlight a few. (i) Electrons can oscillate along the nonparabolic dispersion curve $\epsilon_{k}$ (*7*), within their respective bands. (ii) The electron’s wavefunction can split at degenerate band crossings during one half-cycle of the field. In the subsequent half-cycle, the electrons may interfere with a phase difference caused by the distinct paths they followed. (iii) Electrons can be excited from a lower-lying occupied conduction band (CB) state to an unoccupied CB state via single-photon transitions. These excited electrons are then accelerated by the applied field. (iv) If the electric field is sufficiently strong, electrons may reach the BZ edge and undergo Bloch oscillations (*19*).

We examined more closely the role of Bloch oscillations in the intraband motion that leads to the nonlinear polarization of the medium and subsequent currents. We use semiclassical arguments, as the experimental condition of $\gamma_{\text{DL}}>1$ allows localized wave packets (*25*, *46*). For Bloch oscillations to emerge, the field has to be strong enough to accelerate the electron from the momentum value that corresponds to the Fermi energy to the momentum at BZ edge. For our experimental conditions, the Fermi energy momentum is $k\left(E_{F}\right)=5.5\;\text{rad}/\text{nm}=0.34\;G$ , where $G=2\pi /a_{\text{Ir}}$ , and the BZ edge is at k=8.1rad/nm=0.5G . Thus, the electron needs to gain a critical value of momentum of $k_{c}=2.6\;\text{rad}/\text{nm}=0.16G$ . This momentum gain can be delivered by a pulse of amplitude $E_{0}=$ 0.4 V/Å, and exactly such pulses were used in our experiments.

We conducted an integration of motion for the CB electrons of Ir (see Supplementary Text) and fitted the power-law function to the yield of bulk polarization $P\left(E_{0}\right)$ , as we did for the measurement of the current $J_{0}\left(E_{0}\right)$ . We further discuss the integration method and equivalence of **P** and $J_{0}$ in the Supplementary Text .

We obtained an exponent of 5.2 for the calculated *P* (see fig. S8), which is close to the fits of $J_{0}$ of measured values with exponents of 5.4 and 5.5 (Fig. 3). Moreover, the polarization yield based on Bloch oscillations shows a threshold behavior, with threshold at a point when the amplitude of the field within the pulse allows for the momentum gain at the order of $k_{c}$ . Considering this, one could expect a change in the scaling of $J_{0}\left(E_{0}\right)$ about the field value of $E_{0}=$ 0.4 V/Å (see Supplementary Text). This change could manifest itself in the change of the exponent of the power law or in the measured phase ϕ . However, we do not measure such a behavior. This could be due to the following: First, the polycrystalline nature of our sample as $k_{c}$ naturally depends on the orientation between the laser polarization direction and the crystal axis. Second, the interband transitions within conduction bands, e.g., between bands “2” and “4,” as depicted in Fig. 5 with a pink arrow, might throw electron populations close to the BZ edge, triggering the Bloch oscillations already for lower field strengths. To resolve such details of the electron dynamics, further experimental investigation with time-resolved spectroscopy methods supported with computationally intensive theoretical effort would be necessary. A model involving all possible effects is beyond the scope of this study.

### Summary

Our research demonstrates that light field–driven CEP-dependent current generation is not only limited to dielectrics and semiconductors but also occurs in intrinsic metals, confirming its universality for different material types. We showed that the current yield can be increased by increasing the thickness of the metallic nanolayers. Hence, we non-ambiguously identified that the measured currents originate from the driving of the metal. Furthermore, we anticipate that the yield of the CEP-dependent current is proportional to the number of electrons in the driven system. On the basis of the susceptibility measurement, we confirm the presence of a mechanism distinct from the mechanism responsible for light field–driven currents in dielectrics and semiconductors. Consequently, the ultrafast electronic response is enabled solely by the motion in conduction bands.

It is noteworthy to recognize the similarity to the high harmonic generation process in solids. HHG is a universal phenomenon taking place in many types of materials (*6*, *20*, *47*, *48*), as well as 2D materials like graphene (*47*). Despite its universal occurrence, multiple underlying processes of HHG in solids were identified including interband-interaction harmonics such as ionization-induced, recombination, or Kerr-type harmonics and intraband-origin harmonics (*48*–*50*). Similarly, current generation has been identified across various types of solids, including dielectrics (*4*), semiconductors (*9*, *12*), graphene (*14*), and finally metals, as demonstrated in this work. Thus, a complementary study on high-harmonic emission would help to further elucidate the processes related to the oriented current generation. In conclusion, this research not only extends our understanding of CEP-dependent current generation at picojoule pulse energy levels but also highlights exceptional properties of metallic layers in nonlinear light-matter interactions.

## MATERIALS AND METHODS

### Introduction

Here, we provide detailed measurements and methods related to the phenomenon of ultrafast current generation in samples consisting of Ir and alumina (Al_2_O_3_) layers. This document contains additional data and insights that are closely related to the content of the main text.

In the following section, we show 3D scans of the samples that were used as a basis for measuring the current maximum $J_{0,\text{max}}$ for each sample, as illustrated in Fig. 2 (A and B). This is followed by a detailed explanation of the sample preparation method, with references to the inset of Fig. 1A. Subsequently, we present measurements on the gold sample that are relevant to the power scaling observed in the two samples with the highest Ir content (see Fig. 3).

In the main text, we compare the current yields $J_{0,\text{max}}$ with nonlinear optical characteristic of the samples. Here, we describe in detail the Z-scan method to determine the third-order nonlinear susceptibility of each sample, as shown in table S1 in the Supplementary Text. These measurements led to the identification of an empirical relationship between the measured current maxima $J_{0,\text{max}}$ and $\chi^{\left(3\right)}$ , depicted in Fig. 4.

### 3D scans of samples

**Fig. 6.**
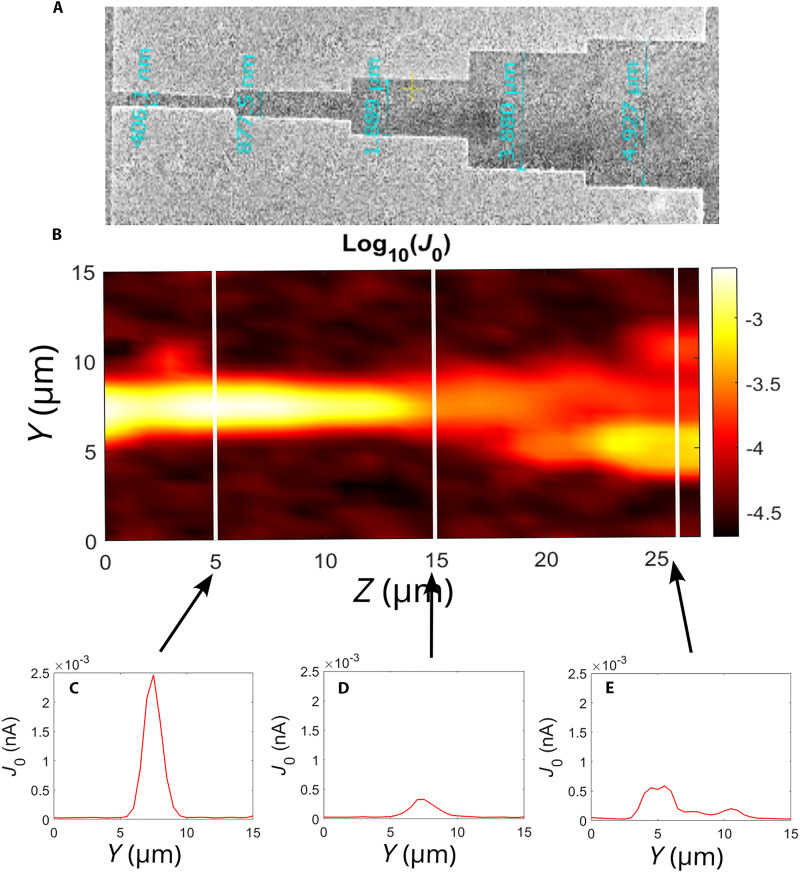
Scanning electron microscopy image and a current map with slices from different Z positions. (**A**) Scanning electron microscopy image of the sample’s junction (widest gap, 5 μm; narrowest gap, 0.4 μm). (**B**) Actual measurement data; a position scan over the junction, where the logarithm of current is visible on the heatmap. The signal copies the geometry of the sample. (**C** to **E**) Slices from the data at selected Z positions, where the current is shown as a function of the horizontal *y* coordinate.

**Fig. 7.**
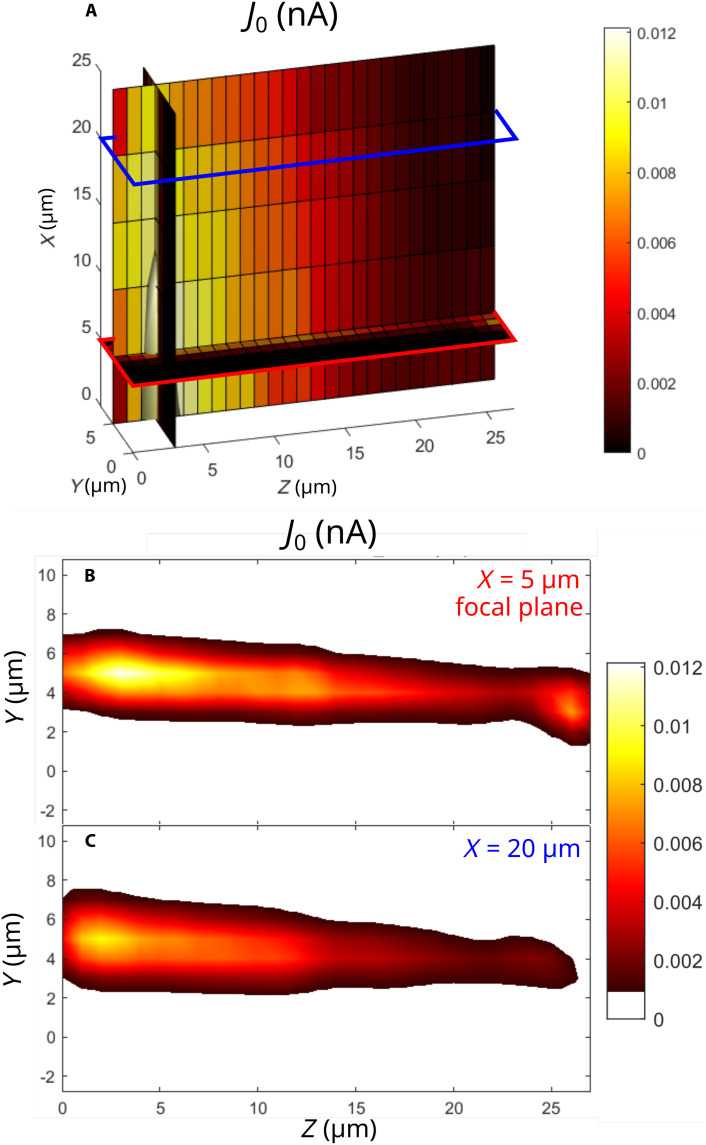
Current maps with dependence on the depth (*X* position). (**A**) Representing the 3D scan with three slices, which intersect in the maximum, where the current reaches its highest value. In this case, we took the highest Ir content sample, which has f=55% iridium content. (**B**) 2D scan fixing the depth (*x* axis, which corresponds to laser propagation direction) in the focal plane, where it reaches the global maximum of the current value, which is 12 pA. (**C**) Out of the focus, we measure lower current values, as we reach a different depth position (*x* axis).

The CEP-dependent current $J_{0}$ has a strong dependence on the illumination geometry. The factor as size of the laser focus, size of the gap between the electrodes, or position of the sample with respect to the electrode influence the magnitude of the current $J_{0}$ in the circuit. To obtain a characteristic magnitude of the CEP-dependent current, we solved the effect of the illumination geometry with two approaches. First, we eliminated the effect of the respective size of the laser focus and the electrode in a way that the sample contains gaps of size smaller, identical, and larger than the laser focal spot size. We implemented step-like electrodes providing five gap sizes in the range of 25 μm (see Fig. 6A). Second, a 3D scan was performed to find a position between the sample and the laser focal volume, which gives the highest signal denoted as $J_{0,\text{max}}$ in the main text. This way, all gaps were probed in multiple focal distances and lateral positions. In Fig. 7, we show the volumetric data of $J_{0}\left(x,y,z\right)$ with slices in selected positions along the optical axis *x*. The *x* axis was varied from 0 to 25 μm. In this case, the focal plane was at 5 μm, where we can see the highest measured current point in Fig. 7B. When we are further away from the focal plane, we measured lower current values as expected (see Fig. 7C).

### Sample preparation

Iridium thin layers are prepared in a matrix of dielectric [samples were prepared by Paul *et al.* (*21*)] of Al_2_O_3_ using the ALD technique (*28*). ALD is a chemical vapor deposition based on sequential and self-limiting surface reactions. Each ALD cycle consists of four sequential steps: precursor pulse [Ir(acac)_3_], precursor purge, co-reactant pulse (O_2_ gas), and co-reactant purge. To better understand the nucleation and layer formation of thin metallic Ir films in thermal ALD processes using the Ir(acac)_3_ precursor, see (*28*).

We have different ALD-grown samples, which differ in the concentration of the iridium, meaning that the volume fraction of iridium is different for different samples. A set of Ir/ Al_2_O_3_ heterostructures was under investigation. On the substrate, gold electrodes were patterned, which were enclosing a tiny gap. These electrodes have a pine tree–shaped step-like structure (typical size, 0.5 to 5 μm).

We have also tested samples containing a gold layer. A layer of 3.5 nm of Au was deposited by an evaporation method on a fused silica substrate. The layer was then capped with 20-nm-thick Al_2_O_3_ layer using an ALD technique.

### Measurement on gold-containing samples

**Fig. 8.**
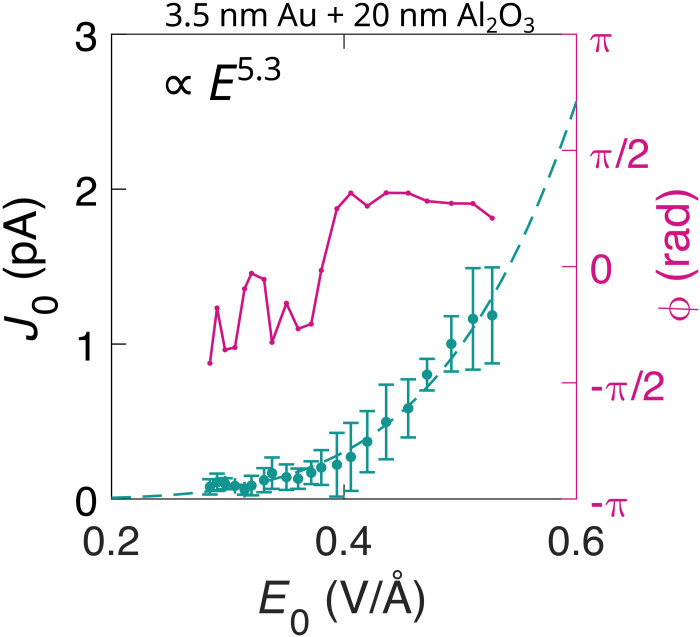
The measured current on the gold nanolayer as a function of the electric field strength. Ultrafast current from a nanolayer of gold of 3.5 nm thickness coated with 20-nm ALD of Al_2_O_3_. Green: Measured current $J_{0,\text{max}}$ as a function of the electric field amplitude $E_{0}$ . Dashed line show a power-law fit yielding an exponent of 5.3. Magenta: Measured phase ϕ.

A sample containing a nanolayer of gold ( $d_{1}=$ 3.5 nm capped with $d_{{\text{Al}}_{2}O_{3}}=$ 20 nm ALD of Al_2_O_3_) was examined similarly as the iridium samples: 3D scan on a range of electrode gaps to obtain the maximum characteristic current $J_{0,\text{max}}$ and power scan to obtain the dependence of the current yield on the electric field amplitude. From 3D scans, we obtained $J_{0,\text{max}}$ = 1.95 pA when exposed to 0.53 V/Å, marking a 13-fold increase in the current when compared to the current of 0.15 pA (obtained at same illumination conditions) of a sample containing an Al_2_O_3_ layer on fused silica without Au. We consider this sample as metallic fraction $f=d_{1}/\left(d_{1}+d_{{\text{Al}}_{2}O_{3}}\right)=15\%$ for the visualization in Fig. 2A. The power scan for the current dependence on the electric field amplitude (see Fig. 8) was fitted with a power law, yielding $J_{0}=E^{5.3}$.

### Modeling the intraband dynamics

The dynamics of the electron within conduction band (neglecting band transitions) can be expressed with the following formalism: It is possible to calculate the equation of motion for the electrons on the Fermi surface in the relevant conduction bands using the Boltzmann equation (*18*, *20*, *51*). Hence, electrons move according to Bloch’s acceleration theorem

$$\hbar \frac{dk}{dt}\left(t\right)=-eE\left(t\right)$$ (2)

where k is the central momentum of an electron wave packet and E(t) is the electric field evolution of the driving laser. In the Coulomb gauge with vector potential defined as $E=-\frac{dA}{dt}$ , the integration of this equation of motion leads to

$$k\left(t\right)=k\left(0\right)-e\frac{A\left(t\right)}{\hbar }$$ (3)

which is an expression to obtain the position of a wave packet in *k*-space in time t based on its initial position k(0) . Namely, it represents a shift in *k*-space, which is given exclusively by the shape of the driving field. The initial distribution of electrons, $f_{k}\left(0\right)$ , is given by the product of the density of states (DOS) obtained from the DFT calculation and Fermi distribution

$$g\left(\epsilon_{k}\right)=\frac{1}{\text{exp}\left(\frac{\epsilon_{k}-E_{F}}{k_{B}T}\right)+1}$$ (4)

where $\epsilon_{k}$ is the energy of an electron of momentum k , $E_{F}$ is the Fermi energy, and $k_{B}T$ is a product of the Boltzmann constant and temperature. Hence

$$f_{k}=\text{DOS}\left(\epsilon_{k}\right)\cdot g\left(\epsilon_{k}\right)$$ (5)

For the distribution in some advanced time f(t) , one simply needs to evaluate *f* at shifted k points, i.e.

$$f_{k}\left(t\right)=f_{k+e\frac{A\left(t\right)}{\hbar }}\left(0\right)$$ (6)

To determine the current j(t) , one needs to calculate the average group velocity of a state f(t) . This is where the shape of the conduction band plays a role as the group velocity $v_{g}$ is defined as

$$v_{k,n}=\frac{1}{\hbar }\nabla_{k}\epsilon_{k,n}$$ (7)

noting that n stands for a number of a band under consideration. Thus, one can calculate the average group velocity in one band by integrating over the momenta of the BZ

$$j_{n}\left(t\right)=-e\frac{1}{V_{\text{cell}}}\int_{\text{BZ}}f_{k}\left(t\right)v_{k,n}\frac{d^{3}k}{4\pi^{3}}$$ (8)

Hence, the total current is a vectorial current sum over the relevant bands. Finally, the polarization *P*(*t*) is calculated by an additional integration

$$P\left(t\right)=\int_{-\infty }^{t}j\left(t^{\prime }\right)dt^{\prime }$$ (9)

The residual polarization is evaluated after the passage of the laser pulse in time t=+∞ , when the laser field is zero

$$P_{t=\infty }\equiv P\left(t\to +\infty \right)$$ (10)

### Third-order susceptibility $\chi^{\left(3\right)}$ measurement using Z-scan technique

The third-order nonlinearity of the Ir/Al_2_O_3_ composite films is measured using the Z-scan technique (*52*). In the measurement, the output of a 10-kHz Ti:sapphire chirped pulse amplification system is used as the laser source, which has a center wavelength of ∼790 nm and a pulse duration of ∼67.15 fs.

## Supplementary Materials

### This PDF file includes:

Supplementary Text Figs. S1 to S7 Tables S1 and S2 References
