# Supplementary material for: Light field–controlled PHz currents in intrinsic metals
Source: Sci Adv. 2025 Jun 25;11(26):eadv5406. doi: 10.1126/sciadv.adv5406 (PMC12189952; doi:10.1126/sciadv.adv5406)
Supplement: Supplementary file 1 — Supplementary Text Figs. S1 to S7 Tables S1 and S2 References [file sciadv.adv5406_sm.pdf]

Science Advances
AAAS

Supplementary Materials for

Light field–controlled PHz currents in intrinsic metals

Beatrix Fehér *et al.*

Corresponding author: Péter Dombi, dombi.peter@wigner.hu

Sci. Adv. **11**, eadv5406 (2025)
DOI: 10.1126/sciadv.adv5406

This PDF file includes:

Supplementary Text
Figs. S1 to S7
Tables S1 and S2
References

Supplementary Text

Introduction

In this supplementary document, we provide detailed analysis related to the phenomenon of ultrafast current generation in samples consisting of Ir and alumina (Al_2O_3) layers. This document contains additional data and insights that are closely related to the contents of the Main manuscript.

Empirical scaling of $J_0(\chi^{(3)})$ compared to theory is the first paragraph of this section. We discuss here the dependence of the measured current on the nonlinear susceptibility.

Based on these experimental results, we proceeded to model the intraband dynamics. We discuss the mechanism of intraband electron motion on a model dispersion curve with the presence of Bloch oscillations Fig. S5 (a–c).

Following on with detailed description of the Z-scan method, we present also the calculations of the penetration depth and a discussion of the field distribution within the samples. The final section of this supplementary information discusses the role of the upper layers in the generation and detection of ultrafast currents.

Empirical $J_0(\chi^{(3)})$ scaling compared to theory

In the Main manuscript, we refer to Khurgin's model that has been used elsewhere for obtaining the current from nonlinear susceptibility $\chi^{(3)}$ (*10,12,41*). We evaluate this model with parameters of our system and obtain rather strong discrepancy with the measurement. This discrepancy is presented in Fig.4 of Main manuscript. In Fig. S3 we show the evaluation for every $\chi^{(3)}$ as a function of the electric field amplitude. Every scaling is fitted with a power-law, providing an exponent for the scaling. One can see that according to this model, the scaling gets steeper with growing $\chi^{(3)}$, which is in contrast to our observation, as we measure a constant exponent for all metallic samples.

Modeling the intraband dynamics

(*This section is a duplication of the section in Main manuscript in Materials and Methods*) Dynamics of electrons within a conduction band (neglecting band transitions) can be expressed with following formalism: It is possible to calculate the equation of motion for the electrons on the Fermi surface

in the relevant conduction bands using the Boltzmann equation (*18, 20, 51*). Hence, electrons move according to Bloch's acceleration theorem

$$\hbar\frac{\mathrm{d}\mathbf{k}}{\mathrm{d}t}(t) = -e\mathbf{E}(t) \tag{S1}$$

with $\mathbf{k}$ being the central momentum of an electron wavepacket and $\mathbf{E}(t)$ is the electric field evolution of the driving laser. In the Coulomb gauge with vector potential defined as $\mathbf{E} = -\frac{\mathrm{d}\mathbf{A}}{\mathrm{d}t}$, the integration of this equation of motion leads to

$$\mathbf{k}(t) = \mathbf{k}(0) - e\frac{\mathbf{A}(t)}{\hbar} \tag{S2}$$

which is an expression to obtain the position of a wavepacket in k-space in time t based on its initial position $\mathbf{k}(0)$. Namely, it represents a shift in k-space which is modulated exclusively by the shape of the driving field. The initial distribution of electrons, $f_{\mathbf{k}}(0)$, is given by the product of the density of states (DOS) obtained from the density functional theory (DFT) calculation and Fermi distribution,

$$g(\epsilon_{\mathbf{k}}) = \frac{1}{\exp\left(\frac{\epsilon_{\mathbf{k}}-E_F}{k_BT}\right)+1}, \tag{S3}$$

where $\epsilon_{\mathbf{k}}$ is the energy of an electron of momentum $\mathbf{k}$, E_F Fermi energy, k_BT is a product of the Boltzmann constant and temperature. Hence,

$$f_{\mathbf{k}} = \mathrm{DOS}(\epsilon_{\mathbf{k}}) \cdot g(\epsilon_{\mathbf{k}}) \tag{S4}$$

For the distribution in some advanced time $f(t)$ one simply needs to evaluate f at shifted $\mathbf{k}$ points, i.e.:

$$f_{\mathbf{k}}(t) = f_{\mathbf{k}+e\frac{\mathbf{A}(t)}{\hbar}}(0). \tag{S5}$$

To determine the current $\mathbf{j}(t)$, one needs to calculate the average group velocity of a state $f(t)$. This is where the shape of the conduction band plays a role as the group velocity $\mathbf{v}$ is defined as:

$$\mathbf{v}_{\mathbf{k},n} = \frac{1}{\hbar}\nabla_{\mathbf{k}}\epsilon_{\mathbf{k},n} \tag{S6}$$

noting that n stands for a number of a band under consideration. Thus, one can calculate the average group velocity in one band by integrating over the momenta of the Brillouin zone:

$$\mathbf{j}_n(t) = -e\frac{1}{V_{\mathrm{cell}}}\int_{\mathrm{BZ}} f_{\mathbf{k}}(t)\mathbf{v}_{\mathbf{k},n}\frac{\mathrm{d}^3\mathbf{k}}{4\pi^3} \tag{S7}$$

Hence, the total current is a vectorial current sum over the relevant bands. Finally, the polarization $P(t)$ is calculated by an additional integration:

$$\mathbf{P}(t) = \int_{-\infty}^{t} \mathbf{j}(t')\mathrm{d}t' \tag{S8}$$

And the residual polarization is evaluated after the passage of the laser pulse in time $t = +\infty$, when the laser field is zero:

$$\mathbf{P}_{t=\infty} \equiv \mathbf{P}(t \longrightarrow +\infty) \tag{S9}$$

The onset of Bloch oscillations: modelling with a test dispersion relation

To capture the importance of the Brillouin zone (BZ) crossing and the subsequent Bloch oscillations we calculated motion of an ensemble of test particles on a test dispersion relation ϵ_k. The test dispersion relation was chosen in a way so that the distance between the E_F and the BZ edge would be similar to iridium system, see Fig. S4(a). Number N of particles was sampled with a uniform distribution $\overline{f}(\epsilon)$ in energy (maximum energy in the system E_F, zero-kelvin approximation). The inversion of the dispersion relation ϵ_k^{-1} was used to obtain $\overline{f}(k)$. Early times in Fig. S4(b) show the initial $\overline{f}(k)$. Next, the temporal evolution $\overline{f}(k,t)$ was obtained by applying principle in eq. (S5) on the distribution $\overline{f}$. If the electric field is high enough this causes the limits of the distribution to reach the BZ edges, hence, the particles change their directions of motion and they are decelerated.

There is two main features that our model shows. The excursion of an accelerated electron to the edge of Brillouin zone (as indicated by arrows in Fig. S4(a,b)) and the onset of the Bloch oscillations as the electron undergoes Brillouin zone (BZ) crossing as soon as it gains normalized momentum higher than 0.5. Here, the normalized momentum is defined as $\overline{k} = k/G$, with $G = 2\pi/a$, a being the lattice constant. In our model, an electron with $\overline{k} > 0.5$ appears in the opposite side of the BZ, i.e., reverses its momentum. Consequently, j current oscillation peaks are being wrapped and strong nonlinearity happens as it can be observed in Fig. S5(b). For iridium such a situation happens when the electric field strength approaches about 0.4 V/Å. Hence, there is a slight discrepancy in the electric field amplitude range in the model and in the experiment as we observe the high increase of the CEP-dependent current (aka the residual polarization) for already lower field amplitudes. Therefore, we think that this might be due to Bloch oscillations from the electrons that underwent an *interband* transition and appeared closer to the BZ edge, see the magenta arrow Fig. S5(a).

Consequently, these electrons might travel to the BZ edge already for lower field strengths. As any *interband* transition are excluded from our modeling, the transitions to the higher conduction bands around $\overline{k} = 0.5$ is not captured either.

The onset of Bloch oscillations: modelling with a dispersion relation obtained from DFT

We applied our modeling of the intraband electron motion on dispersion curves of conduction bands as obtained from a DFT calculations. In the calculation we approximate the evolution of the density function as $f_{\mathbf{k}}(t) = f_{\mathbf{k}+e\frac{A(t)}{\hbar}}(0)$. $A(t)$ is the laser vector potential defined as $\mathbf{A}(t) = -\int_{-\infty}^{t} \mathbf{E}(t')dt'$ with $\mathbf{E}(t)$ defined as in eq. (1) in Main manuscript. As the velocity of the particles is given by $\mathbf{v}_{\mathbf{k},n} = \frac{1}{\hbar}\nabla_{\mathbf{k}}\epsilon_{\mathbf{k}}$, where, $\epsilon_{\mathbf{k},n}$ are the dispersion curves of four relevant bands from DFT, as showed in Fig. S5(a), one can obtain the time evolution of the current density $\mathbf{j}(t)$ and polarization $\mathbf{P}$ according to eq. (S7) and (S8) respectively.

We show such current $\mathbf{j}$ and polarization $\mathbf{P}$ along the laser polarization direction in Fig. S5(b) for a test few-cycle pulse. As the current density cannot follow the sinusoidal shape of the driving field, the medium is left behind with a CEP-dependent residual polarization $P_{t=\infty}(\phi_{\mathrm{CE}})$. This quantity is directly related to the total displaced charge Q and therefore $J_{0,\mathrm{max}}$ as discussed earlier, see eq. (S9). In Fig. S5(b) we show the evolution of $P(t)$ for $\phi_{\mathrm{CE}} = 90°, 0°$.

Furthermore, the amplitude of the CEP modulation of $P_{t=\infty}(\phi_{\mathrm{CE}})$ increases with laser electric field, see Fig. S5(c). Thanks to the simplicity of the model it is now possible to define a sharp boundary in the electric field strength E_0 that marks the onset of Bloch oscillations. This boundary can be understood as a field strength corresponding to the momentum $eA/\hbar$, which bridges the distance between $k(E_F)$ and the Brillouin zone edge: $k/G = 0.5$, where $G = 2\pi/a$. This critical value of momentum along ΓX direction is $k_c/G = 0.16$, which corresponds to about $E_0 = 0.4$ V/Å. k_c naturally depends on the orientation between the laser polarization direction and the crystal axis. We can see in Fig. S5(c) that for low field strengths, where electron motion is purely intraband, the $P_{t=\infty}$ experiences only weak growth with E_0.

As soon as the condition for the Bloch oscillation is fulfilled, i.e., the value of E_0 exceeds 0.4 V/Å, the scaling explodes with power of $m \approx 5$, which is close to the measured values on the high Ir volume fraction samples, see Fig. 3 in Main manuscript, and Au sample in Fig. 8. However, such a sharp change of slope is not observed in the experiment. There can be several reasons

why in the experiment we do not observe such a sharp transition: First, the polycrystalline nature assumes directional averaging of k_c and therefore smearing of such an edge. Second, interband transition, e.g., between bands ”2” and ”4”, as depicted in Fig. S5(a) with a pink arrow, might throw electron populations close to the BZ edge triggering the Bloch oscillations already for lower field strengths. All this electron dynamics will result in a non-trivial shape of the polarization $P(t)$ and will cause nonlinear optical response of the material, represented by a relationship defined in the frequency domain: $P(\omega) = \varepsilon_0 \left(\chi^{(1)} + 3\chi^{(3)}|E|^2\right) E(\omega)$ (*53*). Numerical determination of $P(\omega)$ and $\chi^{(3)}$ characteristic for the system under consideration is, however, out of the scope of this work.

Nonlinear susceptibility of the thin metallic layers

We observe increase of the nonlinear susceptibility as a function of metallic volume fraction f. Theory of an effective medium for waves polarized in the plane of the metallic layers requires that (*54*)

$$\chi^{(3)}_{\mathrm{eff}} = f_{\mathrm{Ir}}\chi^{(3)}_{\mathrm{Ir}} + (1 - f_{\mathrm{Ir}})\chi^{(3)}_{\mathrm{Al_2O_3}} \tag{S10}$$

However, this linear relationship does not reflect the steep growth of the $\chi^{(3)}_{\mathrm{Ir}}$ measured with Z-scan in this work.

Third-order susceptibility $\chi^{(3)}$ measurement using Z-scan technique

A schematic of the Z-scan setup is shown in Fig. S1. The laser is partially picked up by a beam splitter (BS1) and sent into a power meter (PM) for recording a reference intensity, and a focusing lens (FL1) is used to focus the beam onto the sample which is mounted on a motorized linear stage. The sample position is scanned from before focus to after focus while the modulated open aperture (OA) transmittance and closed aperture (CA) transmittance are recorded simultaneously using two photodiodes PD1 and PD2.

Since the Ir/Al_2O_3 composite films are deposited on fused silica (FS) substrate, the FS substrate can actually contribute to the overall Z-scan measurement results (*24*). Therefore, as a reference, a bare FS plate is firstly measured under the same experimental condition with the target samples. The CA and OA transmittance traces of metallic sample (64 cycle, f = 55 %) are shown in Fig. S2.

First, the normalized CA transmittance trace (Fig. S2(a)) shows a dip before the focus ($Z < 0$) which is then followed by a peak after the focus ($Z > 0$), indicating a positive Kerr effect and self-focusing effect in the material originating mostly from the thick FS substrate. Normalized CA transmittance can be fitted with the following formula (*55*):

$$T(x) = 1 + \frac{4x\Delta\Phi}{(1+x^2)(9+x^2)} + \frac{4(3x^2-5)\Delta\Phi^2}{(1+x^2)^2(9+x^2)(25+x^2)} + \frac{32(3x^2-11)x\Delta\Phi^3}{(1+x^2)^3(9+x^2)(25+x^2)(49+x^2)} \tag{S11}$$

where $x = -z/z_0$, z is the longitudinal displacement of the sample from the focus ($z = 0$), $z_0 = \pi\omega_0^2/\lambda$ is the Rayleigh diffraction length, $\Delta\Phi$ is the on-axis phase shift and $\Delta\Phi = (2\pi/\lambda)n_2 I_0 L_{eff}$, λ is the fundamental wavelength, I_0 is the on-axis intensity at the focus, and n_2 is the fitting parameter for Kerr nonlinear refractive index. A n_2 value of ~$8.60954 \times 10^{-21}\ m^2/W$ is obtained from the FS plate, which agrees well with the results reported (*56–58*) and thus could verify the fidelity of our measurement.

Second, the normalized OA transmittance trace (Fig. S2(b)) shows a peak valley at around $Z = 0$. The OA trace can be fitted as:

$$T(x) = \sum_{m=0}^{\infty} \frac{[-q_0]^m}{(m+1)^{\frac{3}{2}}} \tag{S12}$$

$$q_0 = \frac{\beta I_0 L_{eff}}{1+x^2} \tag{S13}$$

where z_0 is the diffraction length of a Gaussian laser beam, β is the nonlinear absorption coefficient, $L_{eff} = (1 - e^{(-\alpha_0/L)})/\alpha_0$ is the sample effective length, α_0 is sample absorption coefficient and L is the sample thickness, both of which can be extracted from the linear measurement results. The determined β value for FS plate is 1.60107×10^{-15} m/W.

Z-scan measurements on Ir/Al_2O_3 samples are then conducted under the same experimental condition, and the recorded CA traces and OA traces are fitted. By subtracting the corresponding reference value for FS substrate, the experimentally determined n_2 and β of Ir/Al_2O_3 composite films with different number of Ir cycles can be obtained. Furthermore, the real part and imaginary part of the third order susceptibility $\chi^{(3)}$ of the samples can be calculated using the following formula in SI units: (*59*)

$$\chi^{(3)}_{Re} = \left(\frac{4}{3}\right) n_0 \epsilon_0 c (n_0 n_2 - k_0 k_2) \tag{S14}$$

$$\chi^{(3)}_{Im} = \left(\frac{4}{3}\right) n_0 \epsilon_0 c (n_0 k_2 - k_0 n_2) \tag{S15}$$

where n_0 and $k_0 = \lambda\alpha/4\pi$ are the linear refractive index and extinction coefficient, $k_2 = \lambda\beta/4\pi$ is the nonlinear extinction coefficient. The results are summarized in Table S1. Here, negative n_2 values correspond to a defocusing characteristic of the sample, and negative β values at higher Ir cycles show a saturable absorption induced by the Ir/Al_2O_3 film.

Field distribution in the sample

Due to the layered structure of the iridium-alumina heterostructure, the electric field distribution in the depth of the sample is not trivial and, moreover, it can differ from one sample to another. Hence, we decided for a simplification and we present the values of the electric field amplitude that is attained at the sample's surface. Here we account for reasons, why we think it is a reasonable representation.

The worst case scenario, when the field does not penetrate deep in the sample is a bulk metal. In that case, the field penetrates into a depth characterized by the evanescent wave. We estimate this length in iridium metal as 19.2 nm at a wavelength of 800 nm, see section below for the derivation. So even in this worst case scenario, this is sufficient for the laser field to act on the heterostructure under consideration. For example, as the laser pulse travels, it first passes through a 3.1-nm-thick layer of Al_2O_3, then it hits the 2-nm-thick layer of Ir (in the case of the 32-cycle iridium sample). In this way, the first metal layer is exposed to only slightly attenuated field compared to the field directly at the entrance to the structure.

For completeness we present the measurement of the transmittance T of the laser beam at low power regime, see Fig. S6 and Table S2. We arrive to values of about T = 0.90 for samples f = 7% to 23% iridium filling fraction and T = 0.83 and T = 0.50 for samples with f = 38% and 55% metal volume fraction respectively.

Estimation of the penetration depth from Drude's theory

The penetration depth calculation is the following. We recall the complex refractive index as:

$$n' = n - i\kappa \tag{S16}$$

The solution of the Helmholtz equation for a plane wave of wave vector $k_0 = \omega/c$ is:

$$E(z,t) = E_0 e^{i\omega t - ik_0 n' z} = E_0 e^{-k_0 \kappa z} e^{i\omega(t - nz/c)} \tag{S17}$$

where z is the coordinate in the direction of the plane wave propagation. Hence, for the decay length of the wave l_{pd} holds true:

$$l_{pd} = \frac{1}{k_0 \kappa} = \frac{c}{\omega \kappa} = \frac{\lambda_0}{2\pi\kappa} \tag{S18}$$

We estimate the refractive index from Drude's theory for metals (*60, 61*):

$$n' = \sqrt{1 - \frac{\omega_p^2}{\omega^2}}. \tag{S19}$$

ω_p is the plasma frequency of free electron gas in metals and it is defined as $\omega_p^2 = \frac{n_e e^2}{\varepsilon_0 m_e}$. The electron density n_e we obtained from the DFT calculation as $n_e^{\mathrm{Ir}} = 7.8 \times 10^{28}$ m^{-3}. For this value and ω corresponding to $\lambda = 800$ nm, the expression under the square root is negative and n' is purely imaginary. Finally, according to eq. (S18) we arrive to the value of penetration depth: 19.24 nm for pure iridium metal.

Importance of top layers in ultrafast current generation and detection

We examined samples that contain multiple layers of iridium. Here, we would like to point out the importance of the top layers in formation of the ultrafast currents from several aspects: 1. Volume fraction has higher influence on the current yield than total thickness of the nanolaminate. 2. Electrodes pick current from top layers more effectively. 3. Notable increase of current with respect to bare dielectric is measurable already for single-metallic-layer samples.

1. For technological reasons, samples of different metal volume fraction had different total thickness (samples differ by number of repetitions of [Ir;Al_2O_3] doublet), see Table S2. From this, one can see that although the total thickness of samples with increasing f (metal volume fraction) decreases (this is true for samples with $f \geq 23\%$), the ultrafast current J_0 increases. At the same time the J_0 grows rather with d_1, which is the thickness of single layers, see Fig. 2 in Main manuscript.

2. The electrode is ohmically insulated from the metallic layer as there is an Al_2O_3 layer between them. Thus, the current in the electrodes has to be generated non-locally as there is no way for electrons to reach the electrodes. This can be explained with Ramo-Shockley theorem that gives a relation for current in a circuit in a vicinity of a moving charge (*8*). In this case, the moving charge is transiently generated as a result of the residual polarization $P_{t=\infty}$ in the metal. The theorem states that the current is proportional to the amount of charge Q moving at velocity v_c, and the weighting field E_{RS}, which is a solution of Poisson equation boundary problem, where the potential of two electrodes is set to 1 and 0 V respectively. In Fig. S7, we show an evaluation (using a finite-element solver (*62*)) of E_{RS} for a 0.4-μm-wide gap between the electrodes. The gold electrodes are 100 nm in height. One can see that E_{RS} drops towards the depth of the sample. Consequently, the current in the circuit must be less sensitive to the charge actuation that happens deeper in the sample.

3. As already presented in Main manuscript section *Measurement on gold-containing samples* and also in Fig. 2 of Main manuscript, the presence of gold and iridium single nanolayers increases substantially the gain of CEP-dependent currents with respect to the gain of currents from bare dielectric, such as Al_2O_3.

These arguments highlight the importance of top metal layers in generation of CEP-dependent currents.

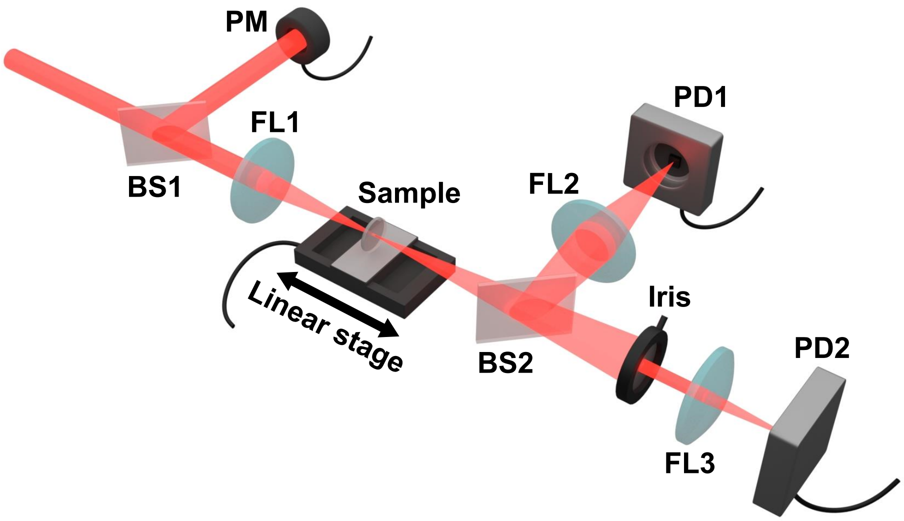

Figure S1: **The Z-scan measurement setup.** For measuring the nonlinear susceptibility. For details see the text.

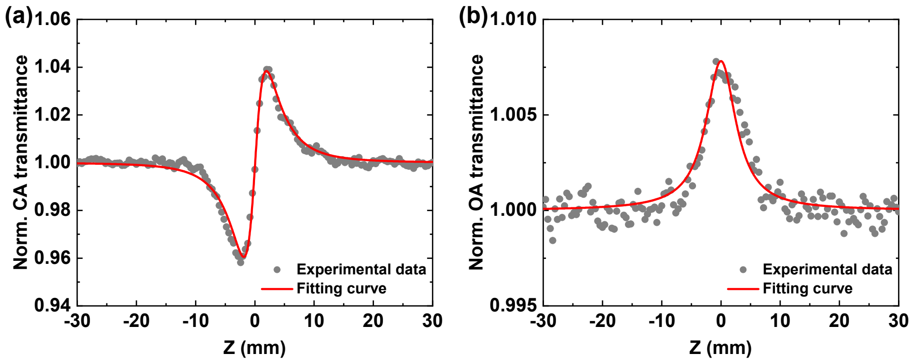

Figure S2: **Normalized transmittance as a function of Z position.** For (a) closed aperture (CA) and (b) open aperture (OA) measurements on the 64 cycle Ir sample, which corresponds to volume fraction of f = 55%.

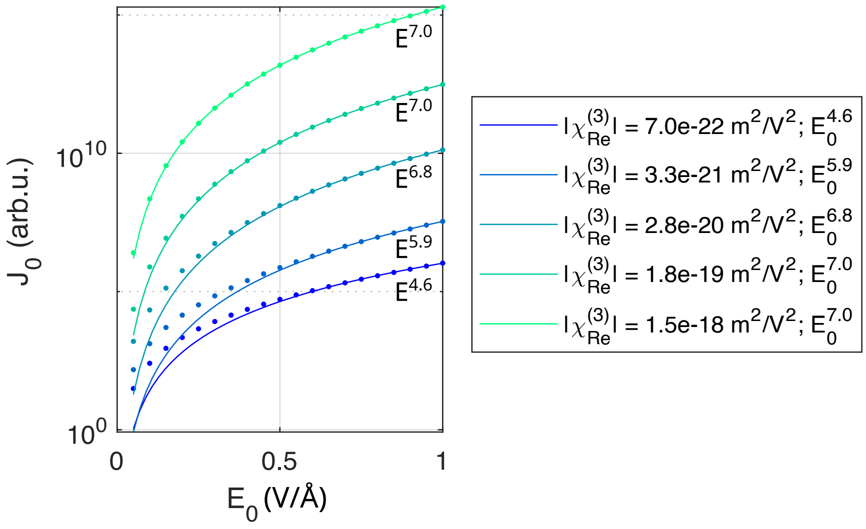

Figure S3: **Amplitude of the CEP-dependent current J_0 as calculated with a virtual carrier model (Khurgin).** Resolved in electric field amplitude E_0 for every measured $\chi^{(3)}$ of studied samples. Exponents obtained from the power law fit are showed.

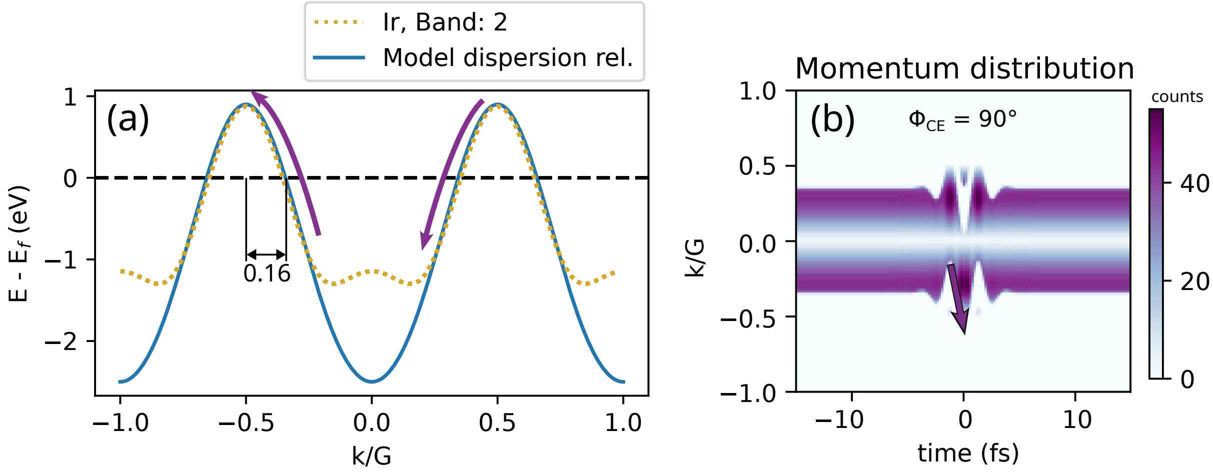

Figure S4: **Calculated iridium $E(k)$ dispersion relation.** (a) (dotted line) Ir $E(k)$ dispersion relation obtained from DFT and simplified model relation (solid). The former corresponds to band "2" presented in the Fig. 4 of the Main manuscript. In Ir case, the distance in k-space between the electron at Fermi surface to the Brillouin zone edge is $k/G = 0.16$. This corresponds to the field of 0.4 V/Å. (b) Distribution of test electrons in k-space as a function of time (b). The electrons are driven by a 2.5-fs laser pulse of $\phi_{\text{CE}} = 90°$. Purple arrows in (a) and (b) sign the motion of the Fermi surface at the time of the strongest half-cycle of the laser field.

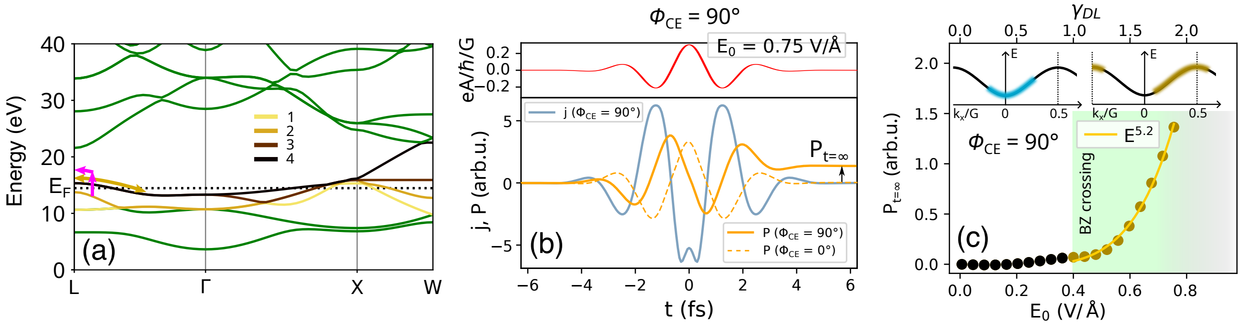

Figure S5: **Band structure and Fermi level of a bulk Ir metal, calculated residual polarization.** (a) Band structure and Fermi level (dotted line) of a bulk Ir metal, calculated with a GPAW code using density functional theory (DFT). Labelled with numbers 1–4 are the bands, that contain Fermi surface. (b) Upper panel shows the momentary vector potential of the laser pulse for a selected value of ϕ_{CE}. Lower panel shows corresponding calculated current (light blue) and polarization (orange). Note the residual polarization $P_{t=\infty}$. (c) Maximum of $P_{t=\infty}(\phi_{\mathrm{CE}})$ as a function of the electric field strength fitted with a power law yields exponent of 5.2. The fitted slope is in a perfect correspondence with the measured values, see Fig. 8. The electron population on an exemplary $\epsilon(k)$ exposed to weak (left inset) and (right inset) strong electric field. Latter shows the Bloch oscillations as the momentum overflows $k/G = 0.5$.

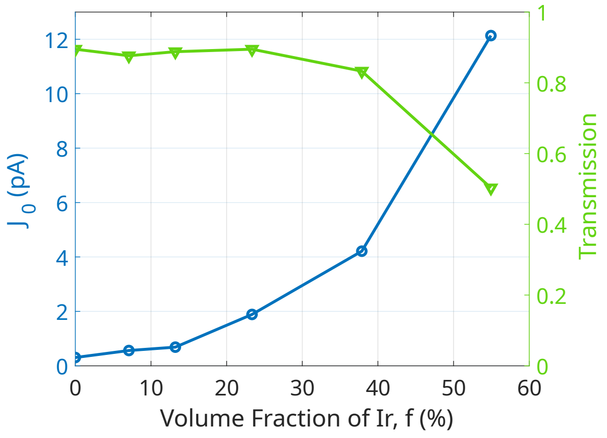

Figure S6: **Measured maximum current and transmittance of the samples.** (blue circles) Measured maximum current (J_0) and (green triangles) transmittance as a function of the volume fraction of the iridium (f).

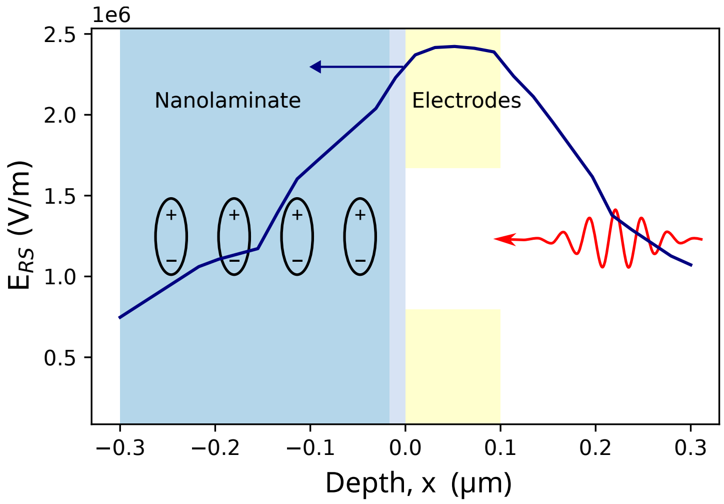

Figure S7: **Calculated weighting field in and above the sample** (blue line) Profile of the calculated weighting field E_{RS} in and above the sample. Shaded squares depict approximately the geometry of situation. Metal electrodes (yellow) are separated with an insulating layer of Al_2O_3 (light blue, not in scale) and metal nanolaminate follows (dark blue). In Poisson solver, the latter was simulated as a homogeneous material with dielectric permittivity higher than air. From right, the laser pulse impinges (red) and creates a transient polarization in the nanolaminate.

Z-scan measurement data				
f (%)	$n_2(m^2/W)$	$\beta(m/W)$	$\chi^{(3)}_{Re}(m^2/V^2)$	$\chi^{(3)}_{Im}(m^2/V^2)$
0	-3.33397E-20	-5.99029E-13	-3.17583E-22	-3.58725E-22
13	-1.54932E-19	8.96796E-14	-1.49389E-21	5.4361E-23
23	-1.33085E-18	1.46832E-12	-1.28323E-20	8.90047E-22
38	-7.52532E-18	-7.74791E-13	-8.06923E-20	-5.22287E-22
55	-4.67341E-17	-3.37853E-11	-6.95585E-19	-3.16127E-20

Table S1: **Z-scan measurement data.** Nonlinear refractive index n_2, nonlinear absorption coefficient β and third order susceptibility $\chi^{(3)}$ of Ir/Al_2O_3 composite films with different Ir cycles.

Sample	Volume Fraction of Ir, f (%)	Max. current, J_0 (pA)	Transmission	Total thickness (nm)
Ir-051	0	0.305	0.895	320
Ir-038	7	0.562	0.877	340
Ir-026	13	0.686	0.888	360
Ir-033	23	1.888	0.895	409
Ir-034	38	4.213	0.833	250
Ir-039	55	12.136	0.503	167

Table S2: **Collection of different properties of the samples.** The sample name and volume fraction of iridium (f) is presented in the first two columns. The measured maximum current ($J_{0,\mathrm{max}}$) and the transmission data is listed in the second pair of columns. The last column is the total thickness.

REFERENCES AND NOTES

1. A. Haché, Y. Kostoulas, R. Atanasov, J. L. P. Hughes, J. E. Sipe, H. M. van Driel, Observation of coherently controlled photocurrent in unbiased, bulk GaAs. *Phys. Rev. Lett.* **78**, 306–309 (1997).

2. R. Atanasov, A. Haché, J. L. P. Hughes, H. M. van Driel, J. E. Sipe, Coherent control of photocurrent generation in bulk semiconductors. *Phys. Rev. Lett.* **76**, 1703–1706 (1996).

3. M. Schultze, E. M. Bothschafter, A. Sommer, S. Holzner, W. Schweinberger, M. Fiess, M. Hofstetter, R. Kienberger, V. Apalkov, V. S. Yakovlev, M. I. Stockman, F. Krausz, Controlling dielectrics with the electric field of light. *Nature* **493**, 75–78 (2013).

4. A. Schiffrin, T. Paasch-Colberg, N. Karpowicz, V. Apalkov, D. Gerster, S. Muehlbrandt, M. Korbman, J. Reichert, M. Schultze, S. Holzner, J. Barth, R. Kienberger, R. Ernstorfer, V. Yakovlev, M. Stockman, F. Krausz, Optical-field-induced current in dielectrics. *Nature* **493**, 70–74 (2013).

5. F. Krausz, M. Stockman, Attosecond metrology: From electron capture to future signal processing. *Nat. Photonics* **8**, 205–213 (2014).

6. S. Ghimire, A. D. DiChiara, E. Sistrunk, P. Agostini, L. F. DiMauro, D. A. Reis, Observation of high-order harmonic generation in a bulk crystal. *Nat. Phys.* **7**, 138–141 (2011).

7. T. T. Luu, M. Garg, S. Y. Kruchinin, A. Moulet, M. T. Hassan, E. Goulielmakis, Extreme ultraviolet high-harmonic spectroscopy of solids. *Nature* **521**, 498–502 (2015).

8. J. Schötz, A. Maliakkal, J. Blöchl, D. Zimin, Z. Wang, P. Rosenberger, M. Alharbi, A. M. Azzeer, M. Weidman, V. S. Yakovlev, B. Bergues, M. F. Kling, The emergence of macroscopic currents in photoconductive sampling of optical fields. *Nat. Commun.* **13**, 962 (2022).

9. O. Kwon, T. Paasch-Colberg, V. Apalkov, B.-K. Kim, J.-J. Kim, M. Stockman, D. Kim, Semimetallization of dielectrics in strong optical fields. *Sci. Rep.* **6**, 21272 (2016).

10. V. Hanus, V. Csajbók, Z. Pápa, J. Budai, Z. Márton, G. Z. Kiss, P. Sándor, P. Paul, A. Szeghalmi, Z. Wang, B. Bergues, M. F. Kling, G. Molnár, J. Volk, P. Dombi, Light-field-driven current control in solids with pJ-level laser pulses at 80 MHz repetition rate. *Optica* **8**, 570–576 (2021).

11. T. Paasch-Colberg, S. Y. Kruchinin, Ö. Sağlam, S. Kapser, S. Cabrini, S. Muehlbrandt, J. Reichert, J. V. Barth, R. Ernstorfer, R. Kienberger, V. S. Yakovlev, N. Karpowicz, A. Schiffrin, Sub-cycle optical control of current in a semiconductor: from the multiphoton to the tunneling regime. *Optica* **3**, 1358–1361 (2016).

12. F. Langer, Y.-P. Liu, Z. Ren, V. Flodgren, C. Guo, J. Vogelsang, S. Mikaelsson, I. Sytcevich, J. Ahrens, A. L'Huillier, C. L. Arnold, A. Mikkelsen, Few-cycle lightwave-driven currents in a semiconductor at high repetition rate. *Optica* **7**, 276–279 (2020).

13. T. Higuchi, C. Heide, K. Ullmann, H. Weber, P. Hommelhoff, Light-field driven currents in graphene. *Nature* **550**, 224–228 (2017).

14. T. Boolakee, C. Heide, A. Garzón-Ramírez, H. B. Weber, I. Franco, P. Hommelhoff, Light-field control of real and virtual charge carriers. *Nature* **605**, 251–255 (2022).

15. N. Altwaijry, M. Qasim, D. Zimin, N. Karpowicz, M. F. Kling, Sensitivity enhancement in photoconductive light field sampling. *Adv. Opt. Mater.* **12**, 2302490 (2024).

16. J. D. Lee, Y. Kim, C.-M. Kim, Model for petahertz optical memory based on a manipulation of the optical-field-induced current in dielectrics. *New J. Phys.* **20**, 093029 (2018).

17. D. C. Mor, Y. Yang, F. Ritzkowsky, F. X. Kartner, K. K. Berggren, N. K. Singh, P. D. Keathley, PHz electronic device design and simulation for waveguide-integrated carrier-envelope phase detection. *J. Lightwave Technol.* **40**, 3823–3831 (2022).

18. M. Ossiander, K. Golyari, K. Scharl, L. Lehnert, F. Siegrist, J. P. Bürger, D. Zimin, J. A. Gessner, M. Weidman, I. Floss, V. Smejkal, S. Donsa, C. Lemell, F. Libisch, N. Karpowicz, J. Burgdörfer, F. Krausz, M. Schultze, The speed limit of optoelectronics. *Nat. Commun.* **13**, 1620 (2022).

19. O. Schubert, M. Hohenleutner, F. Langer, B. Urbanek, C. Lange, U. Huttner, D. Golde, T. Meier, M. Kira, S. W. Koch, R. Huber, Sub-cycle control of terahertz high-harmonic generation by dynamical Bloch oscillations. *Nat. Photonics* **8**, 119–123 (2014).

20. A. Korobenko, S. Saha, A. T. K. Godfrey, M. Gertsvolf, A. Y. Naumov, D. M. Villeneuve, A. Boltasseva, V. M. Shalaev, P. B. Corkum, High-harmonic generation in metallic titanium nitride. *Nat. Commun.* **12**, 4981 (2021).

21. P. Paul, P. Schmitt, V. V. Sigurjónsdóttir, K. Hanemann, N. Felde, S. Schröder, F. Otto, M. Gruenewald, T. Fritz, V. Roddatis, A. Tünnermann, A. Szeghalmi, Atomically thin metal-dielectric heterostructures by atomic layer deposition. *ACS Appl. Mater. Interfaces* **15**, 22626–22636 (2023).

22. V. Hanus, B. Fehér, V. Csajbok, P. Sándor, Z. Pápa, J. Budai, Z. Wang, P. Paul, A. Szeghalmi, P. Dombi, Carrier-envelope phase on-chip scanner and control of laser beams. *Nat. Commun.* **14**, 5068 (2023).

23. M. Hazra, P. Paul, D. Kim, C. David, S. Gräfe, U. Peschel, M. Kübel, A. Szeghalmi, A. N. Pfeiffer, Nonlinear polarization holography of nanoscale iridium films. *New J. Phys.* **25**, 123011 (2023).

24. P. Schmitt, P. Paul, W. Li, Z. Wang, C. David, N. Daryakar, K. Hanemann, N. Felde, A.-S. Munser, M. F. Kling, S. Schröder, A. Tünnermann, A. Szeghalmi, Linear and nonlinear optical properties of iridium nanoparticles grown via atomic layer deposition. *Coatings* **13**, 787 (2023).

25. S. Kruchinin, F. Krausz, V. Yakovlev, Strong-field phenomena in periodic systems. *Rev. Mod. Phys.* **90**, 021002 (2017).

26. M. Wu, S. Ghimire, D. A. Reis, K. J. Schafer, M. B. Gaarde, High-harmonic generation from Bloch electrons in solids. *Phys. Rev. A* **91**, 043839 (2015).

27. P. Földi, M. Benedict, V. Yakovlev, The effect of dynamical Bloch oscillations on optical-field-induced current in a wide gap dielectric. *New J. Phys.* **15**, 063019 (2013).

28. P. Schmitt, V. Beladiya, N. Felde, P. Paul, F. Otto, T. Fritz, A. Tünnermann, A. V. Szeghalmi, Influence of substrate materials on nucleation and properties of iridium thin films grown by ALD. *Coatings* **11**, 173 (2021).

29. F. Ritzkowsky, M. Yeung, E. Bebeti, T. Gebert, T. Matsuyama, M. Budden, R. E. Mainz, H. Cankaya, K. K. Berggren, G. M. Rossi, P. D. Keathley, F. X. Kärtner, On-chip petahertz electronics for single-shot phase detection. *Nat. Commun.* **15**, 10179 (2024).

30. B. Piglosiewicz, S. Schmidt, D. J. Park, J. Vogelsang, P. Groß, C. Manzoni, P. Farinello, G. Cerullo, C. Lienau, Carrier-envelope phase effects on the strong-field photoemission of electrons from metallic nanostructures. *Nat. Photonics* **8**, 37–42 (2014).

31. Y. Yang, M. Turchetti, P. Vasireddy, W. P. Putnam, O. Karnbach, A. Nardi, F. X. Kärtner, K. K. Berggren, P. D. Keathley, Light phase detection with on-chip petahertz electronic networks. *Nat. Commun.* **11**, 3407 (2020).

32. T. Rybka, M. Ludwig, M. Schmalz, V. Knittel, D. Brida, A. Leitenstorfer, Sub-cycle optical phase control of nanotunnelling in the single-electron regime. *Nat. Photonics* **10**, 667–670 (2016).

33. W. P. Putnam, R. G. Hobbs, P. D. Keathley, K. K. Berggren, F. X. Kärtner, Optical-field-controlled photoemission from plasmonic nanoparticles. *Nat. Phys.* **13**, 335–339 (2017).

34. Y. Luo, A. Martin-Jimenez, F. Neubrech, N. Liu, M. Garg, Synthesis and direct sampling of single-cycle light transients by electron tunneling in a nanodevice. *ACS Photonics* **10**, 2866–2873 (2023).

35. J. C. W. Song, L. S. Levitov, Shockley-Ramo theorem and long-range photocurrent response in gapless materials. *Phys. Rev. B* **90**, 075415 (2014).

36. E. N. Osika, A. Chacón, L. Ortmann, N. Suárez, J. A. Pérez-Hernández, B. Szafran, M. F. Ciappina, F. Sols, A. S. Landsman, M. Lewenstein, Wannier-Bloch approach to localization in high-harmonics generation in solids. *Phys. Rev. X* **7**, 021017 (2017).

37. C. Heide, T. Boolakee, T. Higuchi, P. Hommelhoff, Adiabaticity parameters for the categorization of light-matter interaction: From weak to strong driving. *Phys. Rev. A* **104**, 023103 (2021).

38. L. Keldysh, Ionization in the field of a strong electromagnetic wave. *JETP* **20**, 1307 (1965).

39. C. Heide, P. D. Keathley, M. F. Kling, Petahertz electronics. *Nat. Rev. Phys.* **6**, 648–662 (2024).

40. V. Tiwari, B. Gu, I. Franco, Floquet theory and computational method for the optical absorption of laser-dressed solids. *Phys. Rev. B* **108**, 064308 (2023).

41. J. B. Khurgin, Optically induced currents in dielectrics and semiconductors as a nonlinear optical effect. *J. Opt. Soc. Am. B* **33**, C1–C9 (2016).

42. J. J. Mortensen, L. B. Hansen, K. W. Jacobsen, Real-space grid implementation of the projector augmented wave method. *Phys. Rev. B* **71**, 035109 (2005).

43. J. Enkovaara, C. Rostgaard, J. J. Mortensen, J. Chen, M. Dułak, L. Ferrighi, J. Gavnholt, C. Glinsvad, V. Haikola, H. A. Hansen, H. H. Kristoffersen, M. Kuisma, A. H. Larsen, L. Lehtovaara, M. Ljungberg, O. Lopez-Acevedo, P. G. Moses, J. Ojanen, T. Olsen, V. Petzold, N. A. Romero, J. Stausholm-Møller, M. Strange, G. A. Tritsaris, M. Vanin, M. Walter, B. Hammer, H. Häkkinen, G. K. H. Madsen, R. M. Nieminen, J. K. Nørskov, M. Puska, T. T. Rantala, J. Schiøtz, K. S. Thygesen, K. W. Jacobsen, Electronic structure calculations with GPAW: a real-space implementation of the projector augmented-wave method. *J. Phys. Condens. Matter* **22**, 253202 (2010).

44. A. Hjorth Larsen, J. Jørgen Mortensen, J. Blomqvist, I. E. Castelli, R. Christensen, M. Dułak, J. Friis, M. N. Groves, B. Hammer, C. Hargus, E. D. Hermes, P. C. Jennings, P. B. Jensen, J. Kermode, J. R. Kitchin, E. L. Kolsbjerg, J. Kubal, K. Kaasbjerg, S. Lysgaard, J. B. Maronsson, T. Maxson, T. Olsen, L. Pastewka, A. Peterson, C. Rostgaard, J. Schiøtz, O. Schütt, M. Strange, K. S. Thygesen, T. Vegge, L. Vilhelmsen, M. Walter, Z. Zeng, K. W.

Jacobsen, The atomic simulation environment–a Python library for working with atoms. *J. Phys. Condens. Matter* **29**, 273002 (2017).

45. T. Weitz, C. Heide, P. Hommelhoff, Strong-field Bloch electron interferometry for band-structure retrieval. *Phys. Rev. Lett.* **132**, 206901 (2024).

46. S. Longhi, Bloch oscillations and Wannier-Stark localization in a tight-binding lattice with increasing intersite coupling. *Phys. Rev. B* **80**, 033106 (2009).

47. N. Yoshikawa, T. Tamaya, K. Tanaka, High-harmonic generation in graphene enhanced by elliptically polarized light excitation. *Science* **356**, 736–738 (2017).

48. G. Vampa, C. R. McDonald, G. Orlando, D. D. Klug, P. B. Corkum, T. Brabec, Theoretical analysis of high-harmonic generation in solids. *Phys. Rev. Lett.* **113**, 073901 (2014).

49. P. Jürgens, B. Liewehr, B. Kruse, C. Peltz, D. Engel, A. Husakou, T. Witting, M. Ivanov, M. Vrakking, T. Fennel, A. Mermillod-Blondin, Origin of strong-field-induced low-order harmonic generation in amorphous quartz. *Nat. Phys.* **16**, 1035–1039 (2020).

50. H. Liu, Y. Li, Y. You, S. Ghimire, T. Heinz, D. Reis, High-harmonic generation from an atomically thin semiconductor. *Nat. Phys.* **13**, 262–265 (2016).

51. S. Arlinghaus, M. Holthaus, Generalized acceleration theorem for spatiotemporal Bloch waves. *Phys. Rev. B Condens. Matter Mater. Phys.* **84**, 1–11 (2011).

52. M. Sheik-Bahae, A. Said, T.-H. Wei, D. Hagan, E. Van Stryland, Sensitive measurement of optical nonlinearities using a single beam. *IEEE J. Quantum Electron.* **26**, 760–769 (1990).

53. R. W. Boyd, *Nonlinear Optics* (Academic Press, ed. 4, 2020).

54. R. W. Boyd, J. E. Sipe, Nonlinear optical susceptibilities of layered composite materials. *J. Opt. Soc. Am. B* **11**, 297 (1994).

55. X. Zheng, Y. Zhang, R. Chen, X. Cheng, Z. Xu, T. Jiang, Z-scan measurement of the nonlinear refractive index of monolayer WS2. *Opt. Express* **23**, 15616–15623 (2015).

56. D. Milam, Review and assessment of measured values of the nonlinear refractive-index coefficient of fused silica. *Appl. Optics* **37**, 546–550 (1998).

57. M. Grehn, T. Seuthe, W.-J. Tsai, M. Höfner, A. W. Achtstein, A. Mermillod-Blondin, M. Eberstein, H. J. Eichler, J. Bonse, Nonlinear absorption and refraction of binary and ternary alkaline and 30 alkaline earth silicate glasses. *Opt. Mater. Express* **3**, 2132–2140 (2013).

58. T. Olivier, F. Billard, H. Akhouayri, Nanosecond Z-scan measurement of the nonlinear refractive index of fused silica. *Opt. Express* **12**, 1377–1382 (2004).

59. R. del Coso, J. Solis, Relation between nonlinear refractive index and third-order susceptibility in absorbing media. *J. Opt. Soc. Am. B* **21**, 640 (2004).

60. G. Reider, *Photonics: An Introduction* (Springer International Publishing, 2016).

61. Z. Horváth, Fizikai Optika (2016). https://titan.physx.u-szeged.hu/tamop411c/ public_html/ Fizikai%20optika/index.html [accessed: 24 June 2024].

62. M. Malinen, R. Aback, "Elmer finite element solver for multiphysics and multiscale problems" in *Multiscale Modelling Methods for Applications in Material Science*, I. Kondov, G. Sutmann, Eds. (Forschungszentrum Juelich, 2013), chap. Elmer fini, pp. 101–113.
